# *Semen cassiae* Extract Inhibits Contraction of Airway Smooth Muscle

**DOI:** 10.3389/fphar.2018.01389

**Published:** 2018-12-04

**Authors:** Yu-Shan She, Li-Qun Ma, Bei-Bei Liu, Wen-Jing Zhang, Jun-Ying Qiu, Yuan-Yuan Chen, Meng-Yue Li, Lu Xue, Xi Luo, Qian Wang, Hao Xu, Dun-An Zang, Xiao-Xue Zhao, Lei Cao, Jinhua Shen, Yong-Bo Peng, Ping Zhao, Meng-Fei Yu, Weiwei Chen, Xiaowei Nie, Chenyou Shen, Shu Chen, Shanshan Chen, Gangjian Qin, Jiapei Dai, Jingyu Chen, Qing-Hua Liu

**Affiliations:** ^1^Hubei Provincial Key Laboratory for Protection and Application of Special Plants in Wuling Area, Institute for Medical Biology, College of Life Sciences, South-Central University for Nationalities, Wuhan, China; ^2^Lung Transplant Group, Jiangsu Key Laboratory of Organ Transplantation, Department of Cardiothoracic Surgery, Wuxi People’s Hospital, Nanjing Medical University, Jiangsu, China; ^3^Department of Cardiovascular Surgery, Union Hospital, Tongji Medical College, Huazhong University of Science and Technology, Wuhan, China; ^4^Department of Biomedical Engineering, School of Medicine and School of Engineering, University of Alabama at Birmingham, Birmingham, AL, United States; ^5^Wuhan Institute for Neuroscience and Engineering, South-Central University for Nationalities, Wuhan, China

**Keywords:** *Semen cassiae*, *aurantio-obtusin*, airway smooth muscle, relaxation, contraction, ion channels

## Abstract

β_2_-adrenoceptor agonists are commonly used as bronchodilators to treat obstructive lung diseases such as asthma and chronic obstructive pulmonary disease (COPD), however, they induce severe side effects. Therefore, developing new bronchodilators is essential. Herbal plants were extracted and the extracts’ effect on airway smooth muscle (ASM) precontraction was assessed. The ethyl alcohol extract of *semen cassiae* (EESC) was extracted from *Semen cassia*. The effects of EESC on the ACh- and 80 mM K^+^-induced sustained precontraction in mouse and human ASM were evaluated. Ca^2+^ permeant ion channel currents and intracellular Ca^2+^ concentration were measured. HPLC analysis was employed to determine which compound was responsible for the EESC-induced relaxation. The EESC reversibly inhibited the ACh- and 80 mM K^+^-induced precontraction. The sustained precontraction depends on Ca^2+^ influx, and it was mediated by voltage-dependent L-type Ca^2+^ channels (LVDCCs), store-operated channels (SOCs), TRPC3/STIM/Orai channels. These channels were inhibited by *aurantio-obtusin*, one component of EESC. When *aurantio-obtusin* removed, EESC’s action disappeared. In addition, *aurantio-obtusin* inhibited the precontraction of mouse and human ASM and intracellular Ca^2+^ increases. These results indicate that *Semen cassia*-contained *aurantio-obtusin* inhibits sustained precontraction of ASM via inhibiting Ca^2+^-permeant ion channels, thereby, which could be used to develop new bronchodilators.

## Introduction

Approximately 300 million and 328.6 million people suffer from asthma and COPD worldwide (Braman, 2006; Vos et al., 2012). Bronchodilators are a major group of medications for the treatment of these obstructive lung diseases (Dekhuijzen, 2015), however, they cause various side effects such as palpitations, tremor, headache, cardiovascular death (Kajioka et al., 2012), ischemic heart disease, cardiac failure, death due to life-threatening asthma attacks (Abramson et al., 2003; Cazzola et al., 2005; Hasford and Virchow, 2006; Wijesinghe et al., 2009), and desensitization (Nino et al., 2009).

We attempted to screen herbal compounds and develop new bronchodilators. *Semen cassiae* is the seed of *Cassua obtusifolia* L. and *Cassia tora* L., which are globally distributed and used as a medicine and a drink (Zhang et al., 2012). According to Chinese records, “Shennong Ben Cao Jing” (published in approximately 1602∼1616, during the Ming Dynasty), *Semen cassiae* has been used as folkloric medicine to treat various diseases/disorders such as sore red swollen eyes, poor vision, headache, dizziness, diuresis, constipation, and swollen, tinea, and hepatic irrhosis ascites. Later studies have indicated that *Semen cassia* can be used to treat other disorders, for example, neurotoxicity (Ju et al., 2010), diabetic complications (Jung et al., 2010; Fu et al., 2014; Kim et al., 2014), Alzheimer’s disease (Jung et al., 2016), allergy (Kim et al., 2015), and hypertension (Li et al., 2015). Its hypotensive effect led us to investigate whether it has relaxant action in airway smooth muscle (ASM). We found that the ethyl alcohol extract of *Semen cassia* (EESC) inhibits ASM contraction via inhibiting Ca^2+^ permeant ion channels.

## Materials and Methods

### Animals and Ethics Statements

Six-weeks-old male BALB/c mice were purchased from the Hubei Provincial Center for Disease Control and Prevention (Wuhan, China) and were housed in a standard animal facility. All animal use and experimental procedures were approved by the Institutional Animal Ethics Committee of the South-Central University for Nationalities. The license number is 2016-SCUEC-AEC-0030.

### Human ASM

Human bronchial ASMs were from lung tissue samples of transplant donors and recipients and respected tissue from subjects with lung carcinoma. The experiments adhered to guidelines and protocols approved by the Ethics Committee of the South-Central University for Nationalities, and the subjects provided signed, written informed consent.

### Reagents

Nifedipine, acetylcholine chloride (ACh), pyrazole 3 (Pyr3), YM-58483, and agarose were purchased from Sigma (St. Louis, MO, United States). Fluo-4 AM was purchased from Invitrogen (Eugene, OR, United States). *Aurantio-obtusin* was purchased from Yuanye Bio-Technology (Shanghai, China). Nifedipine, Pyr3 and fluo-4 AM were dissolved in DMSO (the final concentration of DMSO ≤ 0.3% in experiments).

### Plant Material and Extraction

*Semen cassiae* was purchased from Beijing TongrenTang (Wuhan, China) and was authenticated by Professor Ding-Rong Wan (College of Pharmacy, South-Central University for Nationalities). A voucher specimen was deposited at the Herbarium of the College of Pharmacy, South-Central University for Nationalities, China. *Semen cassiae* was powdered and then extracted with 70% aqueous ethyl alcohol twice. The extracts were boiled for 1.5 h, and the supernatants were collected and evaporated under reduced pressure. The dried ethyl alcohol extract of *semen cassiae* (EESC) was dissolved in DMSO.

### HPLC Analysis

HPLC analysis was performed using an Ultimate 3000 HPLC system (Thermo Fisher Scientific Inc., United States). The separations were conducted using a ReproSil 100C18 column (4.6 mm × 25 mm, 5 μm; Dr. A. Maisch GmbH, Germany) as the stationary phase and methanol-0.1% aqueous phosphoric acid solution (60:40) as the mobile phase. The flow rate was 1.0 mL⋅min^-1^. The detection wavelength was set at 285 nm.

Semi-preparative HPLC was performed on Agilent 1200 system equipped with a chromatographic column (Elite 5 μm C18 300 Å, 250 mm × 10 mm). The mobile phases consisted of 0.1%H_2_PO_3_ in H_2_O and methanol (the ratio is 30:70). A flow rate of 5 mL/min was used and the chromatograms were monitored at 285 nm. Fractions under the peak area of 25.540 min were separated and removed, the rests were collected (i.e., *aurantio-obtusin*-removed EESC).

To exam the role of *aurantio-obtusin*, which was removed using the semi-preparative HPLC technique (Luo et al., 2018).

### Contraction Measurements of ASM

Contraction of mouse tracheal ASM was measured in epithelium-intact and -denuded tracheal rings (TRs) as previously described, with some modifications (Kajioka et al., 2012; Zhang et al., 2014; Wei et al., 2015; Jiang et al., 2016; Liu et al., 2017). In brief, mice were sacrificed with an intraperitoneal injection of sodium pentobarbital (150 mg/kg). The tracheae were removed and transferred to ice-cold physiological salt solution (PSS) (mM): NaCl 135, KCl 5, MgCl_2_ 1, CaCl_2_ 2, HEPES 10, glucose 10 (pH 7.4). TRs (∼5 mm) were cut from the bottom of the tracheae. Epithelium removal was achieved by gentle mechanical rubbing of the luminal surface with cotton under microscope. TRs were mounted in 10 mL organ baths containing PSS bubbled with 95% O_2_ and 5% CO_2_ at 37°C. A preload of 0.3 g was added. The TRs were equilibrated for 60 min and then contracted with 10^-4^ M ACh three times with an interval of 30 min. After resting for 30 min, experiments were performed. Contraction in human bronchial transverse ASM strips was, similarly, measured, except for the preload was set at 0.3–0.8 g depending on the tissue size.

Contraction of mouse bronchial ASM was also measured in mouse lung slices (Bai and Sanderson, 2006; Jiang et al., 2016). Briefly, mice were euthanized, and the tracheae and lungs were exposed. The tracheae were then cannulated, and each lung was slowly inflated with a warm agarose solution (2%, 37°C, ∼1.3 mL) followed by ∼0.2 mL of air. The lungs were then cooled at 4°C to gel the agarose. Single lobes were removed and sectioned into ∼140 μm thick slices with a vibratome (VT1000S, Leica, Germany). The slices were collected and transferred to Dulbecco’s modified Eagle’s medium (DMEM) (GIBICO) and maintained in an incubator (95%O_2_, 5%CO_2_, 37°C) for 2 h before experiments. The slices were then placed in a chamber and held with a fine nylon mesh, respectively. The chamber was placed under an LSM 700 laser confocal microscope (LSM 700, Carl Zeiss, Goettingen, Germany) and perfused with Hanks’ balanced salt solution (HBSS) at a rate of ∼800 μL/min at room temperature. HBSS contained (in mM) 20 mM HEPES, NaCl 137.93, KCl 5.33, NaHCO_3_ 4.17, CaCl_2_ 1.26, MgCl_2_ 0.493, MgSO_4_ 0.407, KH_2_PO_4_ 0.4414, Na_2_HPO_4_ 0.338, and D-glucose 5.56 and was adjusted to a pH of 7.4. Images under a 10× objective were acquired at a rate of 30 frames/min. The airway lumen areas were measured using Zen 2010 software (Carl Zeiss, Goettingen, Germany).

### Culture of ASM

Mouse TRs were transferred in Dulbecco’s Modified Eagle Medium (DMEM, GIBICO) supplemented with 10% (w/v) fetal bovine serum (FBS, GIBICO), 100 units/mL penicillin and 100 mg/mL streptomycin, and cultured with or without EESC for 24 h at 5% CO_2_ in a 37°C incubator. TRs were washed with PPS and ACh- and 80 mM K^+^-induced contractions were measured.

### Isolation of Single Airway Smooth Muscle Cells

Single mouse tracheal smooth muscle cells were enzymatically isolated as previously described with some modifications (Liu et al., 2009; Wei et al., 2015). Briefly, adult male BALB/c mice were euthanized by intraperitoneal injection of sodium pentobarbital (150 mg/kg). The tracheae were removed and transferred to ice-cold solution containing 136 mM NaCl, 5.36 mM KCl, 0.44 mM KH_2_PO_4_, 4.16 mM NaHCO_3_, 10 mM glucose, 10 mM HEPES, 0.34 mM NaHPO_4_⋅12H_2_O (pH 7.1, adjusted with NaOH). The trachealis tissues were isolated and minced and incubated for 22 min at 35°C in the above solution supplemented with 2 mg/mL papain, 1 mg/mL dithioerythritol, and 1 mg/mL bovine serum albumin (BSA). The tissues were transferred to the above solution supplemented with 1 mg/mL collagenase H, 0.15 mg/mL dithiothreitol, and 1 mg/mL BSA and continued to incubate for 8 min. The tissues were washed and gently triturated in the above solution to release single smooth muscle cells. Cells were stored at room temperature and used for experiments.

### Recordings of Ion Channel Currents

Currents mediated by LVDCCs and ACh-activated channels were recorded using an EPC-10 patch-clamp amplifier (HEKA, Lambrecht, Germany) at room temperature as previously described with some modifications (Tan et al., 2015; Chepurny et al., 2016). Ba^2+^ was used as the charge carrier of LVDCC currents. The intracellular solution contained (in mM) CsCl 130, EGTA 10, MgCl_2_ 4, Mg-ATP 4, HEPES 10, and TEA 10 (pH 7.2, adjusted with CsOH). The extracellular solution contained (in mM) NaCl 105, CsCl 6, BaCl_2_ 27.5, glucose 11, HEPES 10, TEA 10, and NA 0.1 (pH 7.4, adjusted with NaOH). The holding potential was -70 mV, and currents were recorded every 10 s using 500 ms steps from -70 to 40 mV in 10 mV increments. For ACh-activated current measurements, the intracellular solution contained (in mM) CsCl 18, cesium acetate 108, MgCl_2_ 1.2, HEPES 10, EGTA 3, and CaCl_2_ 1 (pH 7.2, adjusted with Tris-HCl). The free Ca^2+^ concentration was approximately 70 nM as calculated using the WEBMAXC standard program^1^. The extracellular solution was PSS without K^+^ containing 10 μM nifedipine, 100 μM NA, and 10 mM TEA to block Ca^2+^, Cl^-^, and K^+^ channel-mediated currents, respectively. Cells were patched in the classical whole-cell configuration and held at -50 mV. Currents were measured using a 500 ms ramp from -80 to 60 mV, and the values at -70 mV were used to represent the current.

### Measurement of Intracellular Ca^2+^

Tracheal smooth muscle cells were loaded with 2.5 fluo-4 AM and the intracellular Ca^2+^ was measured and analyzed as previously described using an LSM 700 laser scanning confocal microscope (Carl Zeiss, Jena, Germany) (Zhang et al., 2014). The background value was subtracted from the fluorescent intensity of cells. The difference values represent the levels of intracellular Ca^2+^.

### Statistical Analysis

The results are expressed as the means ± standard error of the mean (SEM). Student’s *t*-tests between two groups were performed, and statistical significance was established as *P* < 0.05.

## Results

### EESC Inhibits ASM Contraction

**FIGURE 1:**
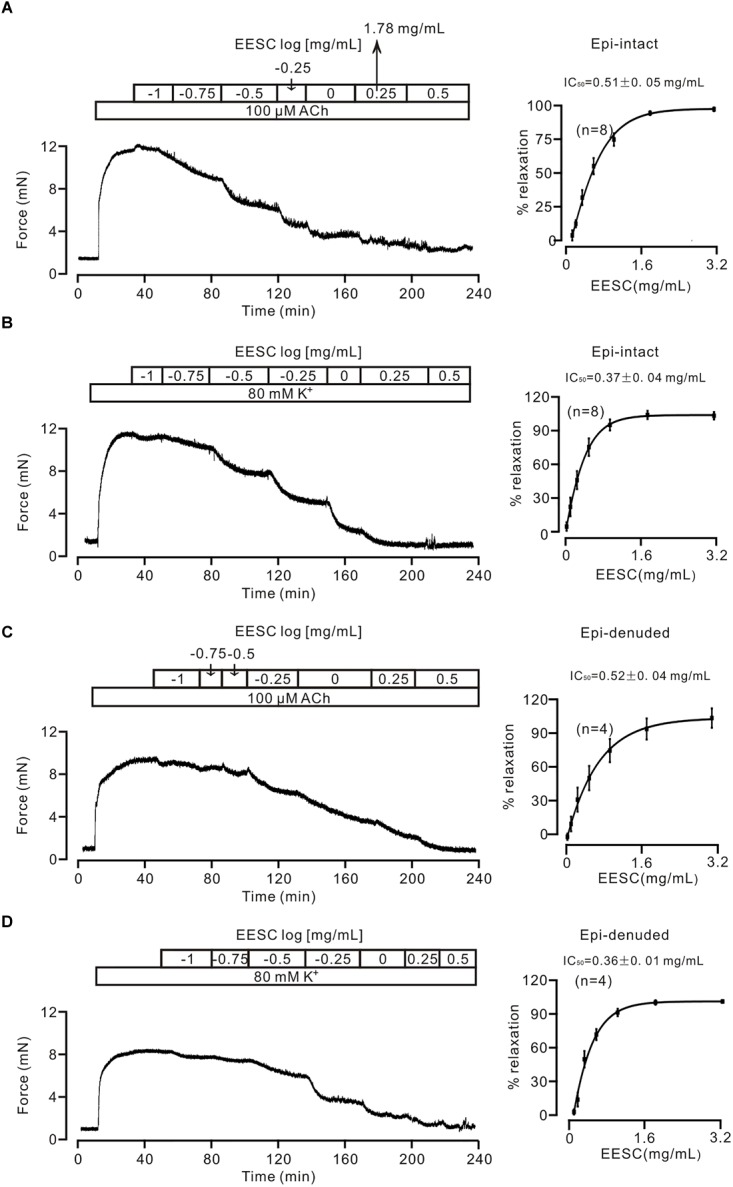
EESC blocks contraction in mouse TRs. **(A)** ACh (100 μM) induced a sustained contraction in a mouse TR, which was blocked by EESC. The dose-inhibition curve is presented. **(B)** Similar experiments were performed, except that the TR contraction was induced by 80 mM K^+^. **(C,D)** The same experiments as above performed in epithelia-denuded TRs. These data indicate that the EESC blocks ASM contraction.

We first tested whether EESC affects the contraction of tracheal smooth muscle. As shown in Figure 1A, a mouse tracheal ring (TR) contracted after application of 100 μM ACh, and this contraction was then decreased by EESC. The dose-inhibition of EESC was shown, and the IC_50_ value and maximal inhibition were calculated as 0.51 ± 0.05 mg/mL and 93.93 ± 1.66% (*n* = 8), respectively. In addition, EESC inhibited the 80 mM K^+^-induced contraction (Figure 1B). The IC_50_ was 0.37 ± 0.04 mg/mL, and the maximal inhibition was 104.24 ± 3.12% (*n* = 8). These data indicate that EESC almost completely blocks ACh- and high K^+^-induced ASM contraction.

We then assessed whether the EESC-induced relaxation was affected by epithelia. The following experiments were performed in epithelia-denuded TRs (Figures 1C,D). The IC_50_ value and maximal inhibition were 0.52 ± 0.04 mg/mL and 104.0 ± 8.7% (*n* = 4), and 0.36 ± 0.01 mg/mL and 102.3 ± 1.1% (*n* = 4) for the precontraction induced by ACh and high K^+^, respectively. These results demonstrate that EESC-evoked relaxation is not mediated by epithelia. Thereby, the following experiments were conducted in epithelia-intact TRs.

**FIGURE 2:**
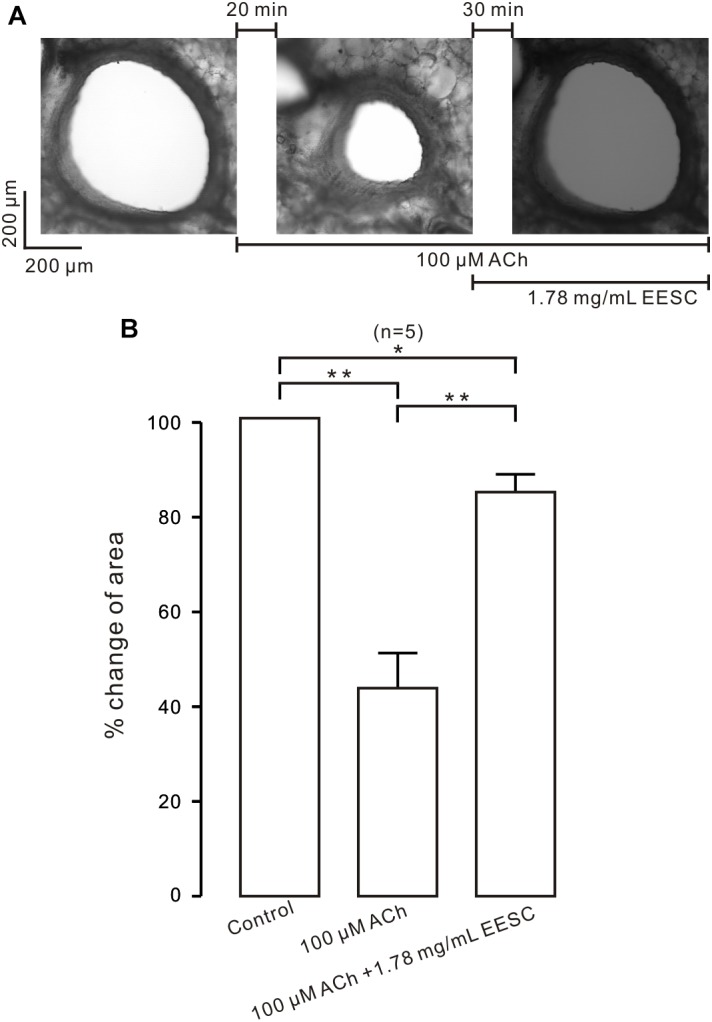
EESC inhibits contraction in lung slices. **(A)** The airway lumen area in a lung slice was decreased by ACh (100 μM) and was markedly increased after the addition of EESC. **(B)** The summary results are shown. ^∗^*P* < 0.05; ^∗∗^*P* < 0.01. These results show that EESC inhibits bronchial ASM contraction.

We next studied whether EESC inhibits contraction in bronchial ASM. As shown in Figure 2A, the lumen area of the airway in a mouse lung slice was observed and was decreased by ACh, and this effect was markedly reversed following application of EESC. However, the reversion was not observed in controls (i.e., in the absence of EESC, data not shown). The summary of the results is shown in Figure 2B. These data indicate that EESC inhibits bronchial ASM contraction.

### EESC-Induced Relaxation Is Due to Inhibition of Ca^2+^-Permeable Ion Channels

**FIGURE 3:**
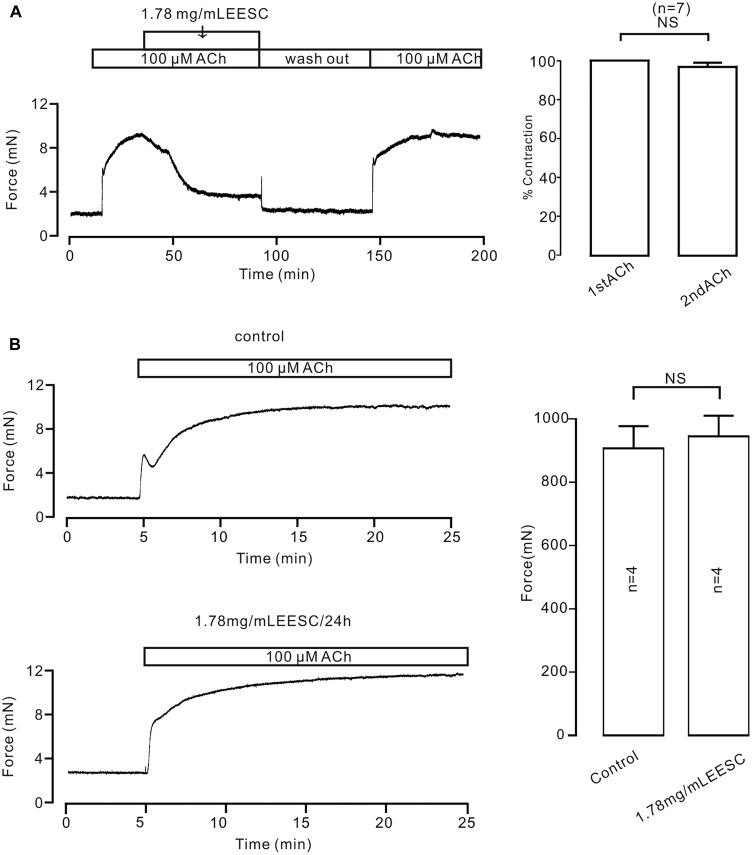
The effect of EESC is reversible. **(A)** ACh induced similar contractions in TRs after washout of EESC. **(B)** ACh-caused contractions were similar in TRs treated with EESC for 24 h and controls. NS: *P* > 0.05. These data suggest that EESC-induced relaxation is not due to EESC-induced cytotoxicity and inhibition of the contraction-related protein expression.

To investigate the underlying mechanism of EESC-induced inhibition on precontraction, we first did two experiments as shown in Figure 3. EESC-induced relaxation is reversible (Figure 3A) and the incubation of 1.78 mg/mL EESC for 24 h did not affect ACh-induced contraction in TRs compared to controls (Figure 3B). These data suggest that EESC-induced relaxation was not due to the cytotoxicity or inhibition on contractile molecules.

**FIGURE 4:**
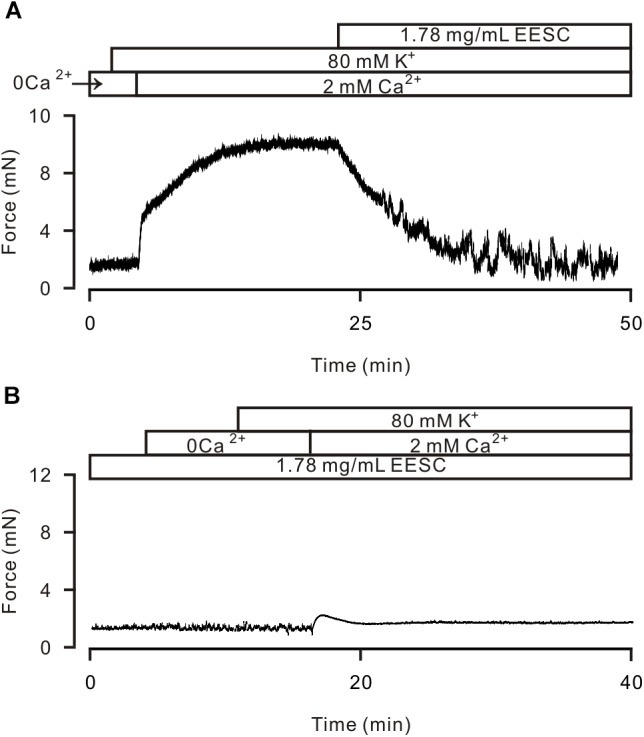
EESC via inhibiting ion channel-mediated Ca^2+^ influx inhibits high K^+^-induced contraction. **(A)** High K^+^ (80 mM) did not trigger contraction in a TR in Ca^2+^-free conditions (0 Ca^2+^ and 0.5 mM EGTA). After the addition of 2 mM Ca^2+^, contraction occurred, which was blocked by EESC. **(B)** After incubation with EESC, the 2 mM Ca^2+^-induced contraction scarcely occurred. Each of the experiments was performed in 8 TRs. These results indicate that EESC-induced relaxation is due to its inhibition of LVDCC-mediated Ca^2+^ influx.

High K^+^ induces activation of LVDCCs, which mediate Ca^2+^ influx and trigger contraction. Thus, we studied whether the EESC-induced relaxation is due to inactivation of LVDCCs. As shown in Figure 4A, 80 mM K^+^ was used to open LVDCCs inducing contraction, however, 80 mM K^+^ did not induce a contraction in TRs in Ca^2+^-free conditions (0 mM Ca^2+^ and 0.5 mM EGTA) but was able to induce a contraction after the restoration of the Ca^2+^ concentration to 2 mM (Figure 4B). The contraction was then completely inhibited by EESC. Furthermore, incubation with EESC almost completely abolished the contraction induced by 2 mM Ca^2+^. These results indicate that EESC-induced relaxation is due to the inhibition of LVDCC-mediated Ca^2+^ influx by EESC.

**FIGURE 5:**
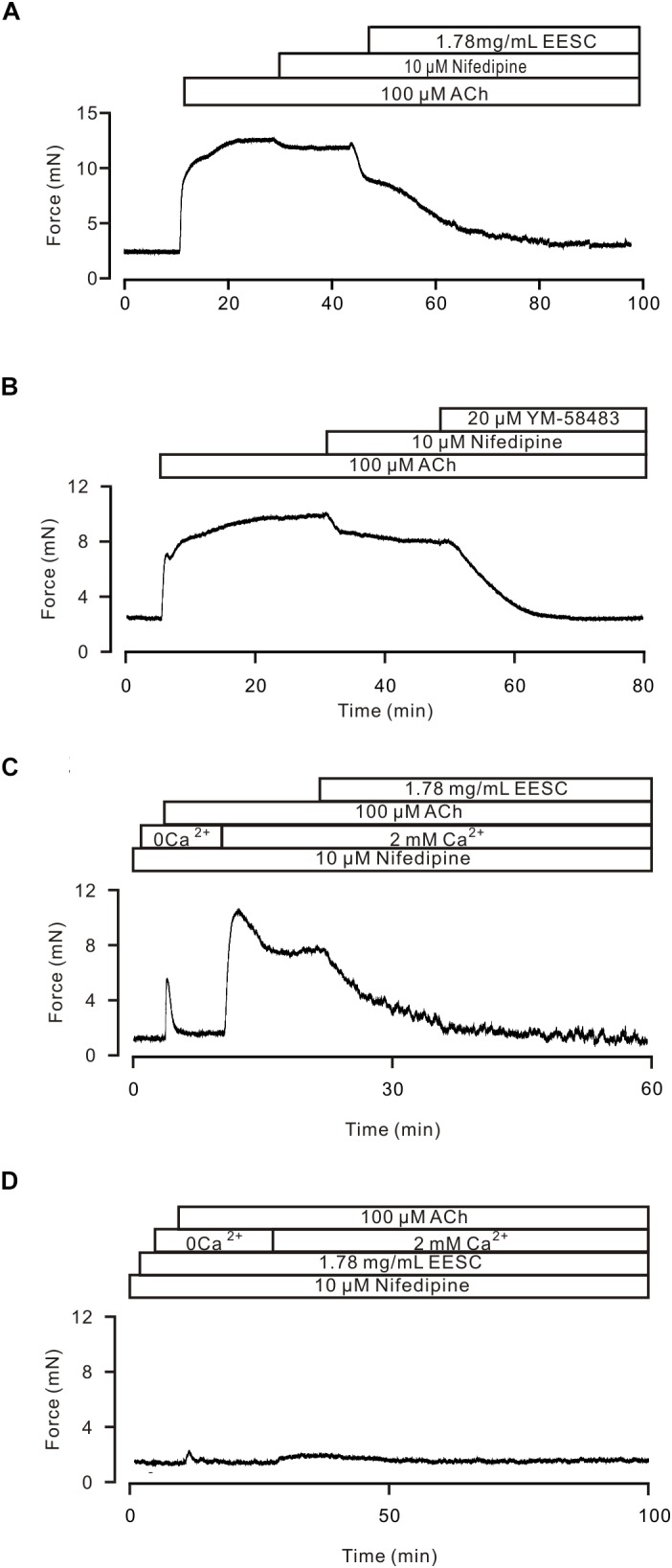
EESC via inhibiting ion channel-mediated Ca^2+^ influx inhibits ACh-induced contraction. **(A)** A TR was contracted by ACh, and the contraction was inhibited by nifedipine and then blocked by EESC. **(B)** The contraction was reduced by nifedipine and then blocked by YM-58483. **(C)** In the presence of nifedipine, ACh induced a transient contraction in Ca^2+^-free conditions (0 Ca^2+^ and 0.5 mM EGTA). After the addition of 2 mM Ca^2+^, a sustained contraction occurred, which was blocked by EESC. **(D)** These contractions were markedly inhibited in an EESC-incubated TR. Each of the above experiments was conducted in 6–8 TRs, and the same results were observed. These results demonstrate that EESC-induced relaxation is due to the inhibition of LVDCCs and other pathways.

We then investigated whether the above mechanism is involved in the EESC-induced inhibition of the ACh-induced contraction. As shown in Figure 5A, ACh-induced contractions in TRs were inhibited with nifedipine, a selective blocker of LVDCCs, and then blocked by EESC. These data suggest that EESC would inhibit LVDCCs and other pathways, thus resulting in relaxation. To define the other pathways, we observed the effect of YM58483, a selective inhibitor of SOCs (store-operated channels) (Ohga et al., 2008) on ACh-induced contraction (Figure 5B), showing that the nifedipine-insensitive contraction was inhibited by YM-58483. The total inhibition was 99.1 ± 2.1% (*n* = 8) by nifedipine and YM-58483. In addition, in the presence of nifedipine and Ca^2+^-free conditions (0 Ca^2+^ and 0.5 mM EGTA), ACh induced a transient contraction, however, a sustained contraction occurred after addition of 2 mM Ca^2+^ and was blocked by EESC (Figure 5C). Both contractions were almost abrogated in EESC-incubated TRs (Figure 5D). These data suggest that other pathways would include Ca^2+^-permeable pathways such as ion channels. Moreover, the transient contraction was blocked by EESC, thereby indicating that EESC resulted in an inhibition of Ca^2+^ release, however, the underlying mechanism was not investigated in this study.

**FIGURE 6:**
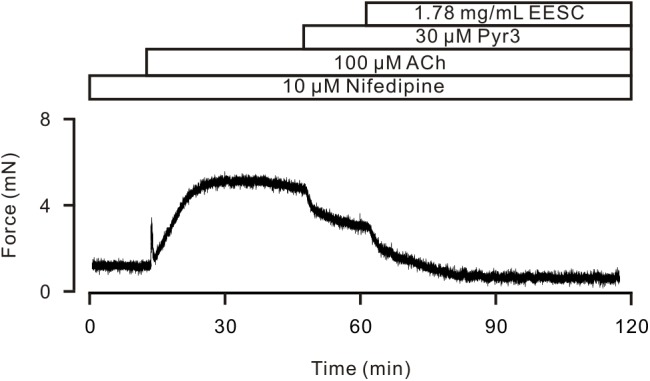
Pyr3 inhibits ASM contraction. ACh induced a sustained contraction in a TR in the presence of nifedipine, which was inhibited by Pyr3 and the remainder was blocked by EESC. This experiment was performed in 6 TRs. These data indicate that EESC-induced relaxation is partially due to the inhibition of Pyr3 sensitive channels.

TRPC3 and STIM/Orai channels have been reported to play a role in the ACh-induced contraction of ASM (Zhang et al., 2014). Therefore, we observed the effects of Pyr3, a selective blocker of these channels. As shown in Figure 6, after LVDCCs were blocked by nifedipine, ACh induced a sustained contraction, which was attenuated by Pyr3 and was further blocked by EESC. These data indicate that EESC-induced relaxation would be partially due to inhibition of TRPC3 and/or STIM/Orai channels.

### *Aurantio-obtusin* Is Included in EESC and Results in Relaxation

**FIGURE 7:**
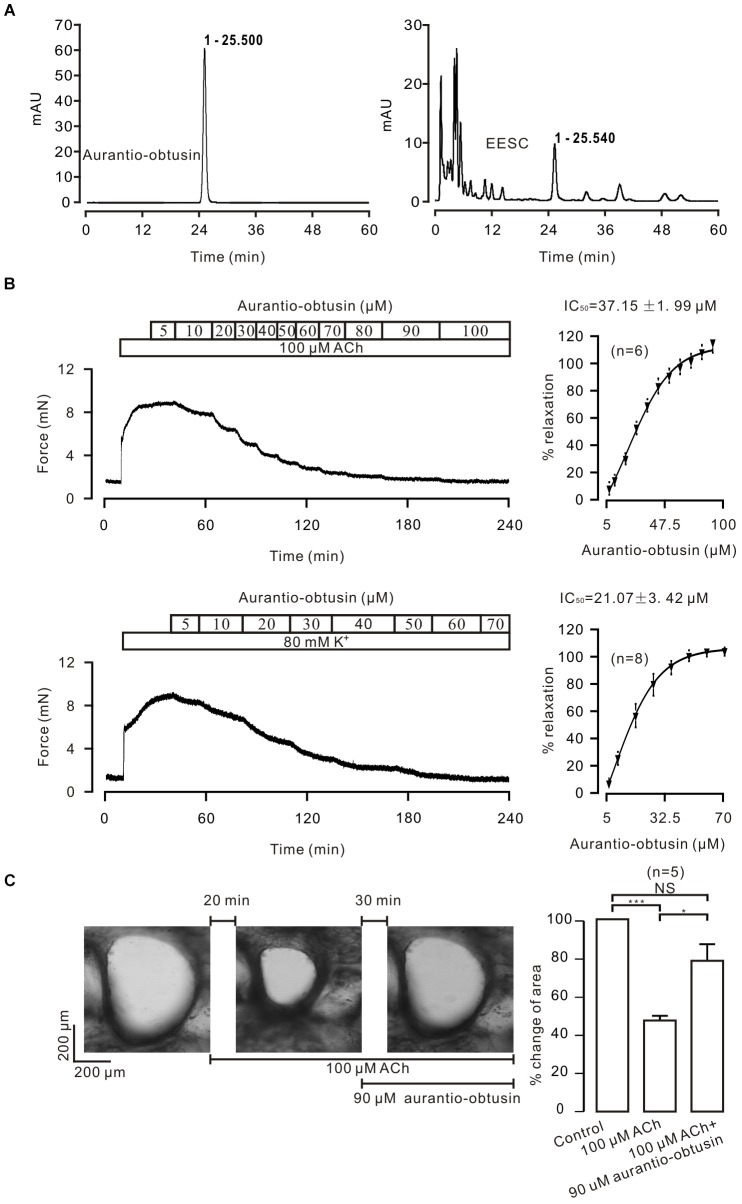
*Aurantio-obtusin* blocks ASM contraction. **(A)** Separation of EESC components and the standard *aurantio-obtusin* were analyzed with HPLC. One peak of EESC overlapped with that of the standard *aurantio-obtusin*. These experiments were conducted twice, and the same results were acquired. **(B,C)** *aurantio-obtusin* blocked the contraction of ASM from TRs induced by ACh and high K^+^, and from lung slices provoked by ACh. These results indicate that *aurantio-obtusin*, which is one component of EESC, inhibits ASM contraction.

To determine which compound is responsible for the EESC-induced relaxation, we separated EESC compounds. S*emen cassiae* has been reported to contain numerous compounds, one of which is *aurantio-obtusin* (Dong et al., 2017; Kwon et al., 2018). As shown in Figure 7A, one peak of EESC (*t_R_* = 25.540) overlapped with that of the standard for *aurantio-obtusin* (*t_R_* = 25.500). These data indicate that *aurantio-obtusin* is present in EESC. We found that the standard *aurantio-obtusin* blocked the contraction of ASM induced by ACh and high K^+^ in mouse TRs (Figure 7B), in mouse bronchi induced by ACh (Figure 7C). These data illustrated that EESC-induced relaxation can be achieved by *aurantio-obtusin*.

**FIGURE 8:**
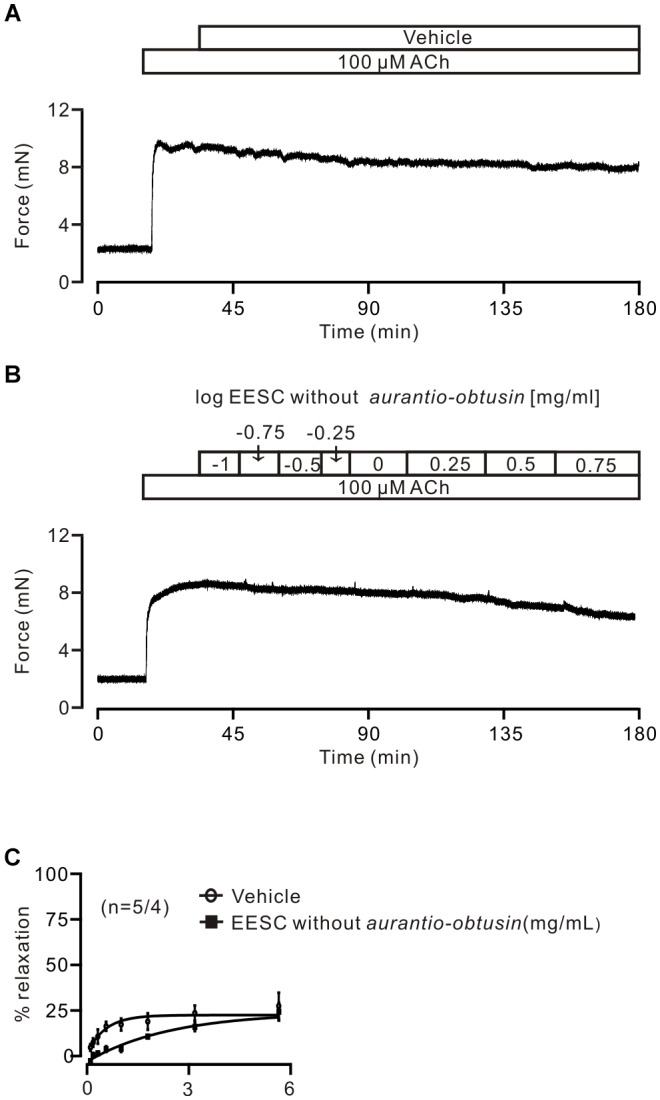
*Aurantio-obtusin-removed* EESC has no effect on ACh-induced contraction. **(A,B)** Representative experiments. **(C)** Dose-inhibition curves of both EESC without *aurantio-obtusin* and vehicle are plotted. These data indicate that the inhibitory action of EESC is achieved by *aurantio-obtusin*.

To further confirm this, *aurantio-obtusin* was removed from EESC and the remaining components failed to induce relaxation compared to the controls (Figure 8).

### *Aurantio-obtusin* Inhibits Ca^2+^ Permeant Ion Channels

**FIGURE 9:**
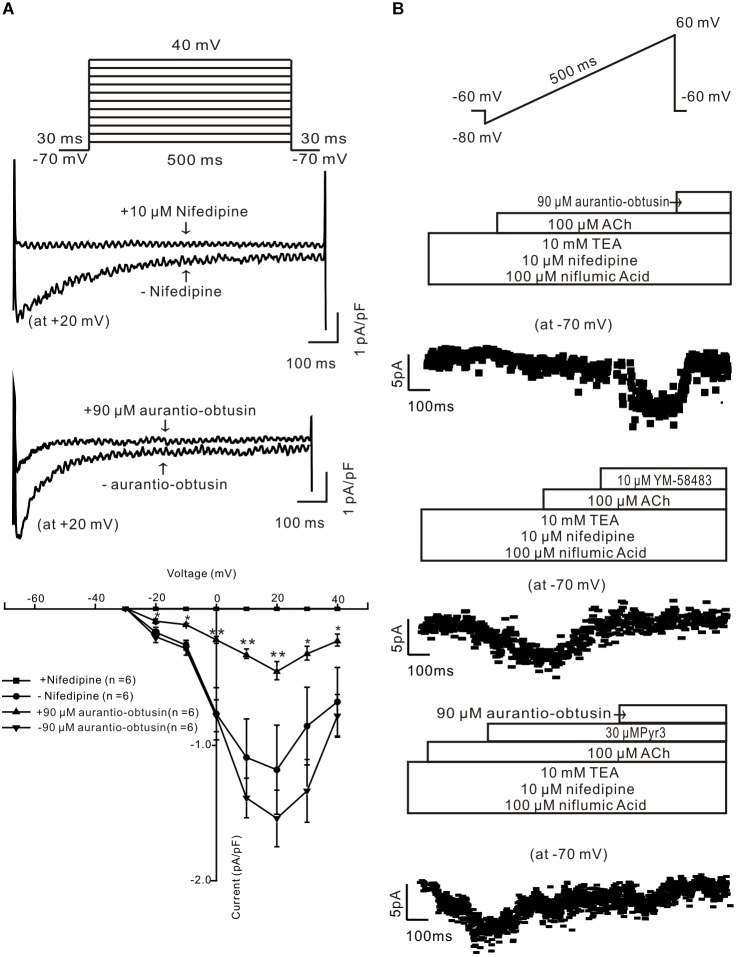
*Aurantio-obtusin* inhibits ion channel-mediated currents in single tracheal smooth muscle cells. **(A)** LVDCC-mediated currents were recorded using the voltage steps (*top*). The representative traces show that the currents are blocked by nifedipine and *aurantio-obtusin* (*middle*). The current-voltage curves were plotted (*bottom*), showing that the currents were completely blocked by nifedipine and markedly inhibited by *aurantio-obtusin*. ^∗^*P* < 0.05; ^∗∗∗^*P* < 0.0001. **(B)** ACh-induced currents were recorded using a ramp (*top*). The non-selective cation channel (NSCC)-mediated currents are isolated in the presence of three blockers. The currents at -70 mV were used to construct time-current traces, showing that *aurantio-obtusin* and YM-58483 completely and Pyr3 greatly inhibited the currents. These results suggest that *aurantio-obtusin* inhibits nifedipine-, YM-58483- and Pyr3-senstive ion channels.

To study the underlying mechanism of *aurantio-obtusin*-induced relaxation, we studied whether *aurantio-obtusin* inhibits LVDCCs and ACh-activated ion channels. LVDCC-mediated currents were measured and which were inhibited by nifedipine and *aurantio-obtusin* (Figure 9A), indicating that *aurantio-obtusin* inhibits LVDCCs. Moreover, *aurantio-obtusin* blocked ACh-induced inward currents from -10.6 ± 2.2 pA to -0.7 ± 0.2 pA (*n* = 6, *P* < 0.0005), by YM-58483 from -10.1 ± 2.4 pA to -0.8 ± 0.3 pA (*n* = 6, *P* < 0.05), and inhibited by Pyr3 from -11.2 ± 3.0 pA to -4.7 ± 2.6 pA (*n* = 6, *P* < 0.05) and further blocked by EESC (Figure 9B). These results indicate that *aurantio-obtusin* inhibits ACh-activated SOCs and TRPC3/STIM/Orai channels.

**FIGURE 10:**
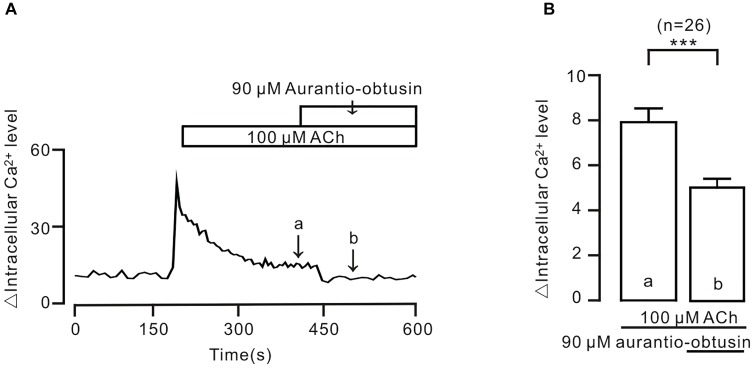
*Aurantio-obtusin* inhibits ACh-induced Ca^2+^ increases in single tracheal smooth muscle cells. **(A)** ACh induced a transient and a sustained increase of intracellular Ca^2+^. The latter was inhibited following the addition of *aurantio-obtusin*. The values at the sites indicated by a and b were obtained. **(B)** Summary results from 26 cells. ^∗∗∗^*P* < 0.001. These results indicate that *aurantio-obtusin* inhibits increased Ca^2+^.

To further confirm the above conclusion, we observed the effect of *aurantio-obtusin* on intracellular Ca^2+^. The result showed that ACh-induced sustained increases of intracellular Ca^2+^ were inhibited by *aurantio-obtusin* (Figure 10), suggesting that the decreases of Ca^2+^ were due to inhibition of above Ca^2+^ permeant ion channels by *aurantio-obtusin*.

### EESC Inhibits Human Bronchial ASM Contraction

**FIGURE 11:**
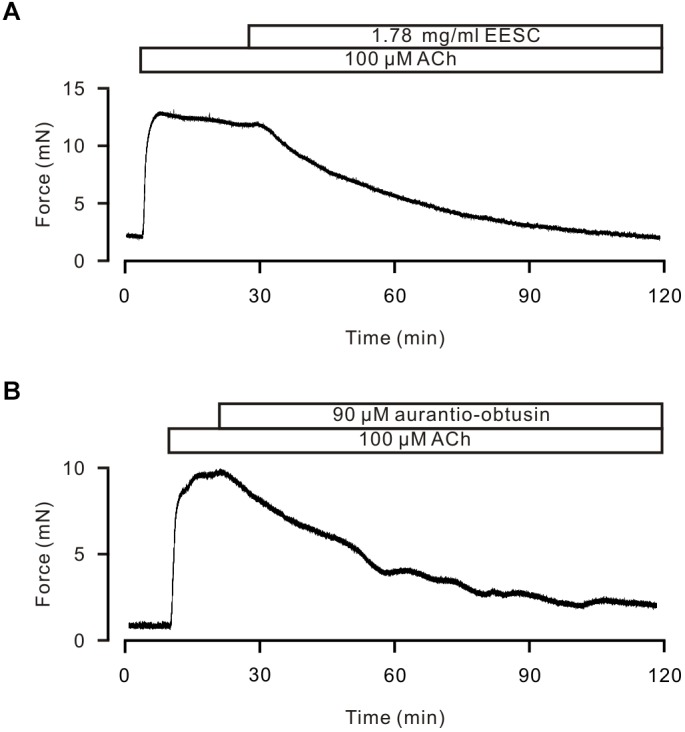
EESC and *Aurantio-obtusin* inhibits ACh-induced Ca^2+^ increases in human ASM. **(A,B)** ACh induced a sustained contraction and was inhibited by EESC and *aurantio-obtusin*, respectively. These results suggest that both have the similar inhibitory action in human ASM.

We finally observed whether EESC and *aurantio-obtusin* has a similar inhibitory effect on human ASM. As shown in Figure 11, human bronchial ASMs were contracted by ACh, and this contraction was inhibited by EESC and *aurantio-obtusin* (*n* = 3 samples from 3 subjects for each experiment).

## Discussion

In this study, we found that the ingredients (i.e., EESC and *aurantio-obtusin*) of *Semen cassia* reversibly reduced both ACh- and high K^+^-induced precontractions in mouse TRs and lung slices. The ACh-induced precontraction was inhibited by the blocker of LVDCCs, SOCs and TRPC3/STIM/Orai channels, respectively. These channel-mediated currents were inhibited by *aurantio-obtusin*, which is one component of EESC. *Aurantio-obtusin* also blocked ACh- and high K^+^-induced precontraction. When a*urantio-obtusin* was removed from EESC using semi-preparative HPLC, EESC failed to induce relaxation. All these results together indicate that EESC-induced relaxant effects resulted from aurantio-obtusin.

The purpose of this study was to screen for active herbal constituents that inhibit ASM contraction. We found that *Semen cassiae* contains such ingredients. Its extract, EESC, potently inhibited ACh- and/or high K^+^-induced contraction of mouse ASM from large and small airways (Figures 1, 2). These results indicate that EESC-induced inhibition of ASM contraction is a general phenomenon, regardless of the differences in ASM location and stimulation type. Furthermore, EESC-induced relaxant effect was independent on epithelium, as EESC produced similar effects on both epi-denuded and epi-intact TRs (Figures 1C,D), suggesting that the NO-related pathway is not involved.

What is the mechanism underlying EESC-induced relaxation? Firstly, this relaxation results neither from the cytotoxicity of EESC, because its inhibition is reversible (Figure 3A) nor from decreased expression of contractile-related molecules, since we did not observe decrease of ACh-induced contraction in TRs treated with EESC for 24 h (Figure 3B).

High K^+^ is known to induce activation of LVDCCs, which then mediate Ca^2+^ influx, subsequently triggering contraction. Our results show that this pathway was blocked by EESC and then resulted in ASM relaxation (Figure 4). Moreover, this effect is achieved by *aurantio-obtusin*, which is one component of EESC (Figure 7A) and blocked LVDCC-mediated contraction (Figure 7B) and currents (Figure 9A).

LVDCCs also involve in ACh-induced precontraction, based on that a selective blocker of LVDCCs, nifedipine, inhibited the contractions (Figure 5A), consistently with previous results (Zhang et al., 2013, 2014). Moreover, these channels are inhibited by *aurantio-obtusin* (Figure 9A). Therefore, EESC-mediated relaxation is partly due to its inhibition of LVDCCs. In addition to LVDCCs, other pathways also involve in EESC-mediated relaxant effect, because EESC further block the contraction in the presence of nifedipine (Figure 5A).

To define the other pathways, we assessed the effect of the selective blocker of SOCs YM-58483 on ACh-induced contraction, since that ACh induces a depletion of Ca^2+^ store, which will then activate SOCs mediating Ca^2+^ influx. The results show that SOC-mediated Ca^2+^ influx plays an important role, because YM-58483 almost blocked the nifedipine-insensitive contraction (Figure 5B). This YM-58483-sensitive Ca^2+^ influx pathway can also be blocked by EESC based on the Ca^2+^ restoration experiments (Figures 5C,D). Moreover, this inhibition is also achieved by *aurantio-obtusin*, because it blocked ACh-induced contractions (Figures 7B,C) and SOC-mediated currents (Figure 9B).

It has been reported that Pyr3 sensitive channels play a role in ACh-induced ASM contraction (Fleischmann et al., 1997; Wang et al., 1997; Zhang et al., 2014). Therefore, we observed the effect of Pyr3, a blocker of TRPC3 and STIM/Orai channels, on ACh-induced contraction. The results showed that Pyr3 partially inhibited the contraction in the presence of nifedipine (Figure 6), thus suggesting that TRPC3 and/or STIM/Orai channels contributed to the contraction. The existence of these channels is further confirmed by that Pyr3 inhibited ACh-induced NSCC-mediated currents (Figure 9B).

It has been reported that *Semen cassiae* contains > 70 chemical compounds including *aurantio-obtusin*. Its aqueous and ethanol extracts have hypotensive effects via inhibiting nervous reflex and iNOS/NO-related receptor-controlled calcium channel-mediated Ca^2+^ influx (Dong et al., 2017). The ethanol extracts include *aurantio-obtusin*, based on our HPLC results (Figure 7A), thus, it will be responsible for above described inhibition of Ca^2+^ influx and relaxation (Dong et al., 2017). This link will be supported by that *aurantio-obtusin* induced relaxation in rat mesenteric arteries, but not pulmonary arteries, through endothelial intracellular PI3K/Akt/eNOS/NO signaling pathway (Li et al., 2015), although this mechanism is different from those above described (Dong et al., 2017).

We further showed that *aurantio-obtusin* induces ASM relaxation via inhibiting increased intracellular Ca^2+^ by blocking ACh-activated Ca^2+^-permeant ion channels, as *aurantio-obtusin* significantly decreased ACh-mediated increasing of intracellular Ca^2+^ (Figure 10), the result was consistent with the previous observation (Dong et al., 2017). Ca^2+^ is a critical modulatory factor for ASM tension and airway inflammation that is a characterized feature of asthma (Kume et al., 2015). Hence, modulating Ca^2+^ dynamics is a remarkable therapeutic target for developing bronchodilators. More recently, a clinical study showed that in patients with severe asthma, LVDCC inhibition attenuated airway remodeling that is another characteristic of asthma (Girodet et al., 2015). In our current study, EESC/*aurantio-obtusin* both exhibited the excellent capability of modulating Ca^2+^ dynamics in ASM. Moreover, both had the similar inhibitory action on human ASM contraction (Figure 11). These results suggest that both can be used to develop potential drugs for asthma and COPD.

In summary, our study demonstrates that EESC-contained *aurantio-obtusin* inhibits ASM contraction through inhibiting Ca^2+^-permeable ion channels. These findings suggest that *aurantio-obtusin* may be used to develop novel antiasthmatic drugs.

## Author Contributions

Y-SS, L-QM, and Q-HL designed the experiments. Y-SS, L-QM, Q-HL, B-BL, W-JZ, J-YQ, M-YL, XL, QW, HX, D-AZ, YYC, X-XZ, LC, JS, Y-BP, PZ, LX, M-FY, WC, XN, CS, ShuC, ShaC, GQ, JD, and JC performed the experiments, analyzed the data. Y-SS, L-QM, and Q-HL wrote the paper.

## Conflict of Interest Statement

The authors declare that the research was conducted in the absence of any commercial or financial relationships that could be construed as a potential conflict of interest.

## Footnotes

1 http://www.stanford.edu/~cpatton/webmaxc/webmaxcS.htm

## Abbreviations

ACh: acetylcholine chloride
ASM: airway smooth muscle
COPD: chronic obstructive pulmonary disease
EESC: ethyl alcohol extract of *semen cassia*
HPLC: high performance liquid chromatography
LVDCCs: voltage-dependent L-type Ca^2+^ channels
Pyr3: pyrazole 3
SOCs: store-operated channels
STIM: stromal interaction molecule
TRPC3: transient receptor potential channel 3
TRs: tracheal rings
